# Preclinical evidence and possible mechanisms of cardioprotective effects of resveratrol in diabetic cardiomyopathy: a systematic review and meta-analysis

**DOI:** 10.1186/s13098-024-01512-8

**Published:** 2024-11-17

**Authors:** Xiaodan Yan, Youjia Hu, Shuyuan Zhao, Qian Zhou, Qiu Chen

**Affiliations:** 1 Hospital of Chengdu University of Traditional Chinese Medicine, Chengdu, China; 2 Chengdu University of Traditional Chinese Medicine, Chengdu, China

**Keywords:** Resveratrol, Diabetic cardiomyopathy, Animal model, Oxidative stress, Meta-analysis

## Abstract

**Introduction:**

Diabetic cardiomyopathy (DCM) is a significant complication of diabetes, characterized primarily by the development of heart failure in individuals with diabetes. Numerous animal studies have indicated that resveratrol enhances cardiac function in diabetic cardiomyopathy; however, its reliability and underlying mechanism remain unclear. This study aims to assess the cardioprotective effects of resveratrol on DCM and explore its potential mechanism.

**Methods:**

We searched PubMed, EMBASE, WOS, Cochrane Library, CNKI, CBM, Chinese VIP, and Wan Fang Database until March 31st, 2024, without language restrictions. Continuous outcome measures were analyzed using weighted mean difference or standardized mean difference, and heterogeneity was assessed with I^2^. The risk of bias in animal experiments was evaluated using the SYRCLE tool, and evidence reliability was determined with the GRADE tool. All data were analyzed using Review Manager 5.4.1 and Stata 17. This study has been registered on the PROSPERO (CRD42024523944).

**Results:**

A total of 18 studies meeting the criteria were identified. The analysis revealed that the resveratrol intervention group exhibited significant improvements in LVEF (WMD = 17.88), LVFS (WMD = 8.77), HW/BW (SMD=-2.92), SOD (SMD = 4.53), and MDA (SMD=-5.07) compared to the control group. The GRADE grading assessment indicated moderate certainty for LVEF, HW/BW, and MDA, while certainty for other factors was considered low.

**Conclusion:**

Our research suggests that resveratrol may protect cardiac function in DCM through anti-inflammatory and anti-oxidative stress effects. However, these findings are based on preclinical data, and further extensive trials are needed to confirm their effectiveness and safety before clinical application.

**Supplementary Information:**

The online version contains supplementary material available at 10.1186/s13098-024-01512-8.

## Introduction

Diabetic cardiomyopathy (DCM), alternatively referred to as diabetic myocardial disorder, represents a pathophysiological state emanating from diabetes. It is distinguished by myocardial dysfunction and structural irregularities, ultimately culminating in heart failure, even in the absence of hypertension, coronary artery disease, or any other cardiac valve ailments [1]. The European Society of Cardiology (ESC) Heart Failure Association and Myocardial and the Pericardial Diseases Working Group has proposed a new definition of diabetic cardiomyopathy in its latest consensus statement for 2024, describing it as a dysfunction in heart contractility and relaxation that occurs in the presence of diabetes [2]. Cardiovascular disease is the leading cause of death among diabetic patients, and mitigating the risk of cardiovascular events in individuals with diabetes has emerged as a pivotal objective in diabetes care and management [3]. Cardiovascular disease is the leading cause of death among diabetic patients, and mitigating the risk of cardiovascular events in individuals with diabetes has emerged as a pivotal objective in diabetes care and management [3]. Diabetic cardiomyopathy has emerged as a significant contributor to the development of heart failure and subsequent patient mortality, irrespective of other risk factors. substantialregardless [4–6].

Early manifestation of DCM is characterized by myocardial fibrosis and increased stiffness, leading to subsequent development of left ventricular hypertrophy, cardiac remodeling, and impaired diastolic function. It ultimately leads to reduced ejection fraction and the onset of heart failure [7]. DCM involves intricate metabolic pathways in its pathogenesis [8]. DCM The current clinical management of DCM primarily relies on conventional pharmacotherapy to regulate blood pressure, reduce heart rate, and manage hyperglycemia rather than providing individualized solutions for diabetes patients with concurrent heart failure [10]. Traditional treatment methods cannot meet the current status of cooccurrence and development in clinical practice rather exploration is needed to discover more efficient preventive and therapeutic approaches [11].

The latest research has demonstrated the favorable impact of plants and their extracts on diabetic cardiomyopathy. Resveratrol (RES), a natural polyphenolic compound derived from various phytology such as blueberry(*Vaccinium spp.*) and grape(*Vitis vinifera* L.), is scientifically known as 3,5,4’-trihydroxy-trans-stilbene [12]. RES’s blood glucose control efficacy has been extensively validated through numerous clinical trials [6, 13, 14]; however, investigation into the cardioprotective effects against DCM is currently limited to animal studies, with the underlying mechanisms yet to be elucidated. Multiple studies have demonstrated that RES exhibits antioxidant effects, enhances vascular relaxation, suppresses angiogenesis, mitigates inflammation and cell apoptosis, effectively safeguards cardiomyocytes, and may improve the cardiac condition of DCM through a variety of mechanisms, fcit [15–19]. Research on RES’s role in DCM has mainly involved animal models and in vitro studies, with limited human clinical trials. Thus, its mechanisms and effectiveness in humans are unclear. Given the lack of robust clinical evidence, we conducted a comprehensive meta-analysis of animal studies to investigate RES’s efficacy and mechanisms.

Our study aimed to summarize the preclinical evidence on the cardioprotective effects of RES in DCM and elucidate the underlying mechanisms, providing a systematic and empirical evidence base for subsequent clinical studies.

## Methods

This manuscript was finalized with the assistance of the Cochrane Handbook [20] and encompasses all necessary components outlined in the PRISMA 2020 checklist [21]. The comprehensive PRISMA checklist can be found in Supplementary file 1. This study has been registered on the PROSPERO platform (CRD42024523944).

### Search strategy

**Fig. 1:**
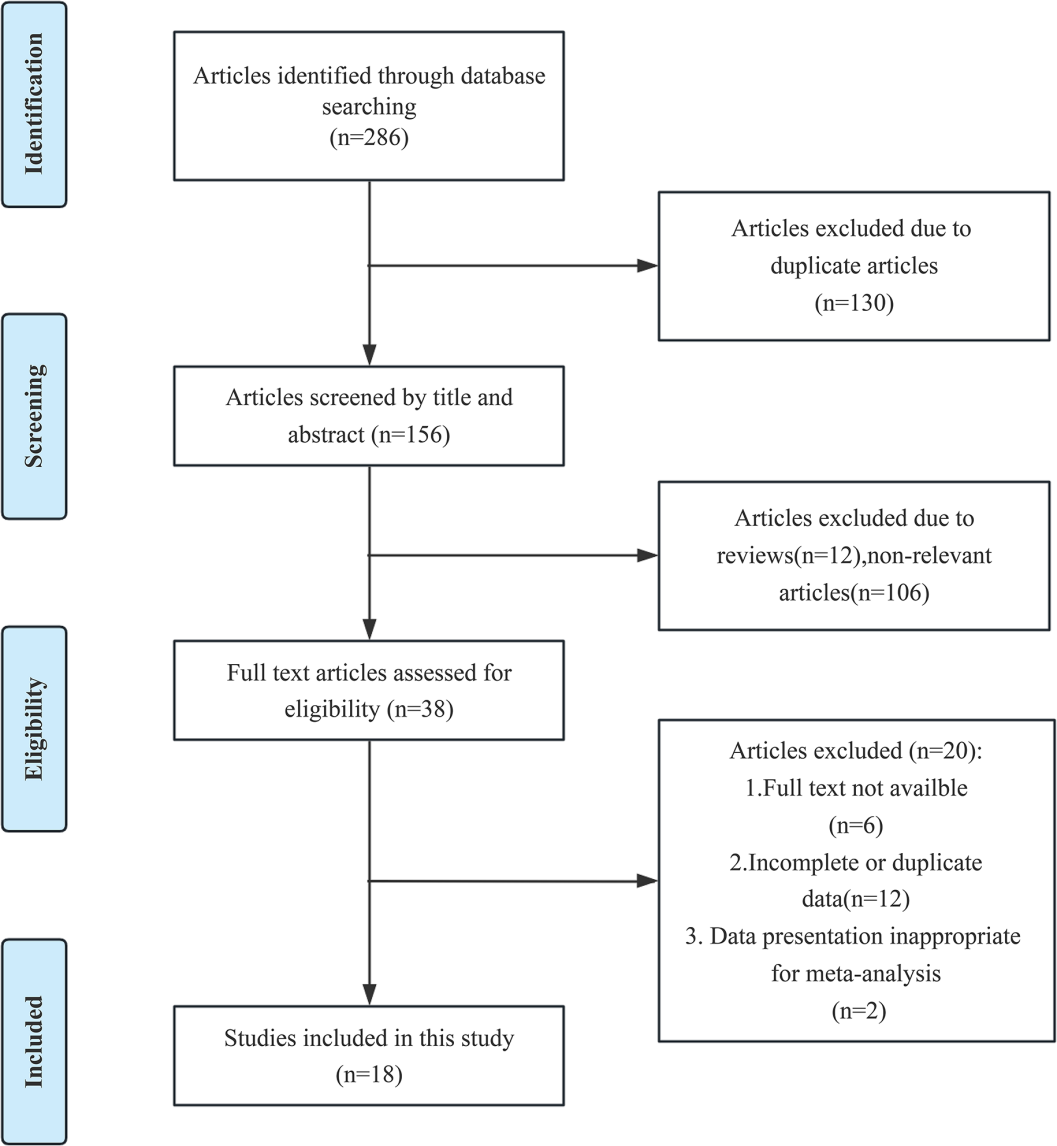
The flowchart of the article screening

Two researchers comprehensively analyzed data from eight databases (PubMed, EMBASE, Web of Science, Cochrane Library, Chinese National Knowledge Infrastructure, Chinese Biomedical Literature Database, Chinese VIP Database, and WanFang Database) until March 31st, 2024. There is no language restriction. The search strategy employed the following terms: (“RES” OR “Vitis vinifera”) and (“Diabetic cardiomyopathy” OR “Diabetic Cardiomyopathies”) for English databases; while for the Chinese database, it was “bai li lu chun” (meaning RES) and “tang niao bing xin ji bing” (meaning Diabetic cardiomyopathy). We implemented adjustments for different databases according to their characteristics. The detailed search formula can be found in Supplementary file 2. The disagreement between the two researchers was resolved through team discussions, leading to a consensus. The flowchart of article screening is illustrated in Fig. 1.

### Selection criteria

The eligibility criteria for the study were as follows: (i)Subjects: Animal models of DM(Diabetes Mellitus) or DCM; (ii) Intervention: The treatment group is exposed to RES as a single therapy, with no restrictions on dosage, drug type, administration route, or duration of treatment; (iii) Comparison: The control group consists of untreated controls or placebo controls; (iv)At least one of the outcome indicators had been reported: Cardiac dysfunction and cardiac hypertrophy measures: Left Ventricular Ejection Fraction (LVEF), Left Ventricular Fractional Shortening Rate (LVFS), Ratio of peak E to peak A (E/A), Heart weight to body weight (HW/BW); Oxidative stress measures: Superoxide Dismutase(SOD), Malonaldehyde(MDA). SOD, an antioxidant enzyme, exhibits antioxidative properties. MDA is a three-carbon aldehyde derived from the free radicals of polyunsaturated fatty acids, indicating lipid oxidation levels [22].

We excluded specific studies based on the following criteria: (i) In vitro studies or clinical trials; (ii) Not written in Chinese or English; (iii) Duplicate data; (iv) Only providing abstracts without full text; (v) Incomplete experimental data.

### Data extraction

Two researchers independently extracted the following data. Our initial approach was to extract numerical data from tables, text passages, or numeric values provided in the study. In cases where such data was not reported explicitly, we first tried to contact the article’s author for access. When unavailable, we utilized digital ruler software (Engauge Digitizer) to retrieve information from charts. Furthermore, the highest dose group and the final time point were selected if there were numerous dose groups and multiple measurement time points.

The following information was extracted from the eligible studies independently: (i) Study ID (first author’s name and publication year); (ii) Number of animals in the RES intervention group and control group for the DM/DCM model, along with the modeling methods employed; (iii) Dosage and administration route of the intervention; (iv) Duration of the intervention; (v) Primary and second outcome measures.

### Quality assessment

We used the SYRCLE animal experiment risk of bias assessment tool to assess the quality of the included studies [23]. Two independent authors evaluated studies separately, and a third author was consulted to decide in case of disagreement. Items will be assessed in three categories: low risk of bias, unclear risk of bias, and high risk of bias. Furthermore, we employ the GRADE tool (Grading of Recommendations Assessment Development and Evaluation) to evaluate the credibility of evidence, which is categorized into three tiers: low, moderate, and high [24].

### Statistical analysis

Due to variations in the experimental designs of animal studies, a random effects model is utilized to combine effect sizes. The weighted mean difference (WMD) method compares continuous variables when measurement methods and units are consistent across different studies; otherwise, the standardized mean difference (SMD) method is utilized. The findings are the average and standard deviation derived from converting the median and quartiles. Furthermore, 95% confidence intervals (95% CI) are computed with statistical significance at *P* < 0.05. Heterogeneity among included studies is assessed using I^2^, whereby significant heterogeneity is indicated when I^2^ exceeds 75%. We performed a meta-regression analysis and subgroup analysis considering various factors, including sample size(≥ 20 or < 20), animal species (mice or rat), specific strains (SD rats, Wistar rats, FVB mice, Cardiac-Specific SIRT1 Knockout mice, or C57/BL6J mice), methods of model establishment (simple STZ injection or STZ injection combined with a high-fat diet or other methods), sex of the animal models (male, female, or unclear), administration route, dosage, and duration of intervention to assess the influence of these variables on the study outcomes. The effect of publication bias was explored via funnel plot or Egger’s test. Data analyses were performed by review manager 5.4.1 and Stata 17.

## Result

### Results of the literature search and characteristics of the studies

This meta-analysis comprises 18 studies [18, 19, 25–40] and includes 363 animals (181 experimental and 182 control). Eight articles are written in Chinese [33–40], while the rest are in English [18, 19, 25–32]. The sample size ranged from 8 to 36 per study. Regarding species, rats accounted for 57.6% (209/363) of the animals, while mice accounted for 42.4% (154/363). In more detail, SD rats accounted for 53.2% (193/363), Wistar rats accounted for 4.4% (16/363), C57/BL6J mice accounted for 18.7% (68/363), FVB mice accounted for 9.9% (36/363), Spontaneous T2DM C57BLKS/J db/db mice accounted for 8.3% (30/363), and the Generation of the Cardiac-Specific SIRT1 Knockout (SIRT1 KO) mice accounted for 5.5% (20/363). Regarding sex, male animals accounted for the majority (77.1%, 280/363), while female animals accounted for 9.1% (33/363), and the remaining 13.8% of individuals (50/363) were not specified as male or female. The modeling methods were a high-fat diet combined with streptozotocin (STZ) intraperitoneal injection or STZ intraperitoneal injection alone. The diverse dosages of RES administered at levels ranging from 2.5 to 400 mg/kg/d and the duration of interventions exhibited variability, spanning from as short as five days up to seven months; however, it is worth noting that one study omitted reporting the specific intervention duration. Table 1 presents the characteristics of the included studies.

**Table 1:** Basic characteristics of included studies

Study ID (author, year)	Species (sex)	sample	intervention	control	Weight	Model (method)	Intervention group (method)	Control group (method)	Duration of treatment	Outcomes
Canbolat, İ P.,2020	SD rats(male)	12	6	6	200–220 g	DM, by by intraperitoneal injection of streptozocin	Treated with Resveratrol (ip,10 mg/kg/d)	Equal volume Saline(ig)	4 weeks	MDA
Diao, J.,2019	SD albino rats(male)	20	10	10	100–150 g	DCM, by high-fat diet(4weeks)and streptozotocin intraperitoneal injection(30 mg/kg).	Resveratrol dissolved with dimethyl sulfoxide and administered (ig,10 mg/kg/d)	Equal volume Saline(ig)	8 weeks	LVEF, LVFS
Dong, H.L.,2013	SD rats(male)	16	8	8	180–220 g	T2DM, by high-sucrose-fat diet (8weeks)and a single intraperitoneal injection of STZ(35 mg /kg)	Treated with resveratrol (ig,40 mg /kg)	Carboxymethylcellulose sodium	6 weeks	SOD, MDA
Du, H.X.,2017	Spontaneous T2DM C57BLKS/J db/db mice(unknown)	30	15	15	50±5 g	SpontaneousT2DM C57BLKS/J db/db mice	Treated with resveratrol (ig,400 mg/kg/d)	Untreated	12 weeks	SOD
Fang, W. J.,2018	SPF SD rats(male)	8	4	4	150±20 g	DM, by by feeding the rats a high-fat diet and injecting them with low-dose STZ (35 mg/kg intraperitoneally)	Treated with Resveratrol (po,50 mg/kg/d)	Untreated	16 weeks	LVEF, LVFS, E/A, HW/BW, SOD, MDA
Huang, A.Q.,2017	SD rats(male)	24	12	12	-	T1DM, by a single intraperitoneal injection streptomycin(65 mg/kg)	Treated with resveratrol (ig,50 mg/kg/d)	Equal volume dimethyl sulfoxide(ig)	8 weeks	LVEF, LVFS, HW/BW
Liu, G.J.,2018	SD rats(male)	10	5	5	150 ± 20 g	T2DM, by high-sucrose-fat diet (12weeks)and a single intraperitoneal injection of STZ(30 mg /kg)	Treated with resveratrol (ig,100 mg/kg/d)	Untreated	12 weeks	LVEF, LVFS, SOD, MDA
Ma, S.,2017	Cardiac-Specific SIRT1 Knockout (SIRT1 KO )mice(unknown)	20	10	10	-	DCM, by intraperitoneally injected with streptozotocin (150 mg/kg /day, for seven consecutive days)	Treated with resveratrol (ip,25 mg/kg/d)	Untreated	5 days	LVEF, LVFS
Mohammadshahi, M.,2014	SD rats(male)	24	12	12	300–350 g	DM, by injection of streptozotocin (50 mg/ /kg/ip)	Resveratrol in aqueous solution(po,5 mg/kg was regulated every week)	Untreated	4 months	SOD
ShamsEldeen, A. M.,2019	Wistar rats(female)	16	8	8	200–230 g	DM, by a single injection of streptozotocin (55 mg/kg/ip)	Treated with Resveratrol (ip,2.5 mg/kg/d)	Untreated	-	LVEF, LVFS, SOD, MDA
Song, X.,2020	SD rats(male)	20	10	10	160–170 g	DCM, by high-sucrose-fat diet(8weeks)and intravenous administration of STZ (30 mg/kg)	Treated with Resveratrol (15 mg/kg)	Untreated	8 weeks	HW/BW, SOD, MDA
Tan, X.T.,2018	C57/BL6J mice(male)	36	18	18	25–30 g	T1DM, by intraperitoneal injection streptomycin(50 mg/kg/day, for five consecutive days)	Treated with resveratrol (ip,7.5 mg/kg/day)	Equal volume Saline(ip)	12 weeks	LVEF, LVFS
Wang, G.,2018	FVB mice(male)	36	18	18	-	T1DM, by intraperitoneally given multiple low doses of streptozotocin (40 mg/kg /day, for fve consecutive days)	Treated with Resveratrol (po,10 mg/kg/d)	Untreated	6 months	LVEF, LVFS
Wang, W.Q.,2023	C57/BL6J mice(male)	20	10	10	22–26 g	DCM, by intraperitoneal injection streptomycin(50 mg/kg/day, for five consecutive days)	Treated with resveratrol (ig,25 mg/kg/d)	Equal volume dimethyl sulfoxide(ig)	12 weeks	LVEF, LVFS, MDA
Wu, Z. Y.,2017	SD rats(male)	12	6	6	250–300 g	T1DM, by a single intravenous injection of STZ (50 mg/kg)	Treated with Resveratrol (ig,50 mg/kg/d)	Untreated	8 weeks	LVEF, LVFS, HW/BW
Wu, B.,2020	C57/BL6J mice(male)	12	6	6	-	DM, by injection STZ citrate buffer solution (55 mg/kg, for five consecutive days)	Treated with resveratrol (ip,50 mg/kg/d)	Untreated	7 days	LVEF, LVFS
Wu, G.X.,2019	SPF SD rats(male)	30	15	15	250–300 g	DCM, by a single intraperitoneal injection streptomycin(60 mg/kg)	Treated with resveratrol (ig,50 mg/kg/d)	Equal volume Saline(ig)	10 weeks	LVEF, LVFS, HW/BW
Yan, R.,2016	SD rats(female)	17	9	8	247±19 g	DCM, by a single intraperitoneal injection of streptozotocin (65 mg/kg)	Treated with trans-Resveratrol (ip,2.5 mg/kg/d)	Untreated	7 months	LVEF, LVFS

### Risk of bias assessment

**Fig. 2:**
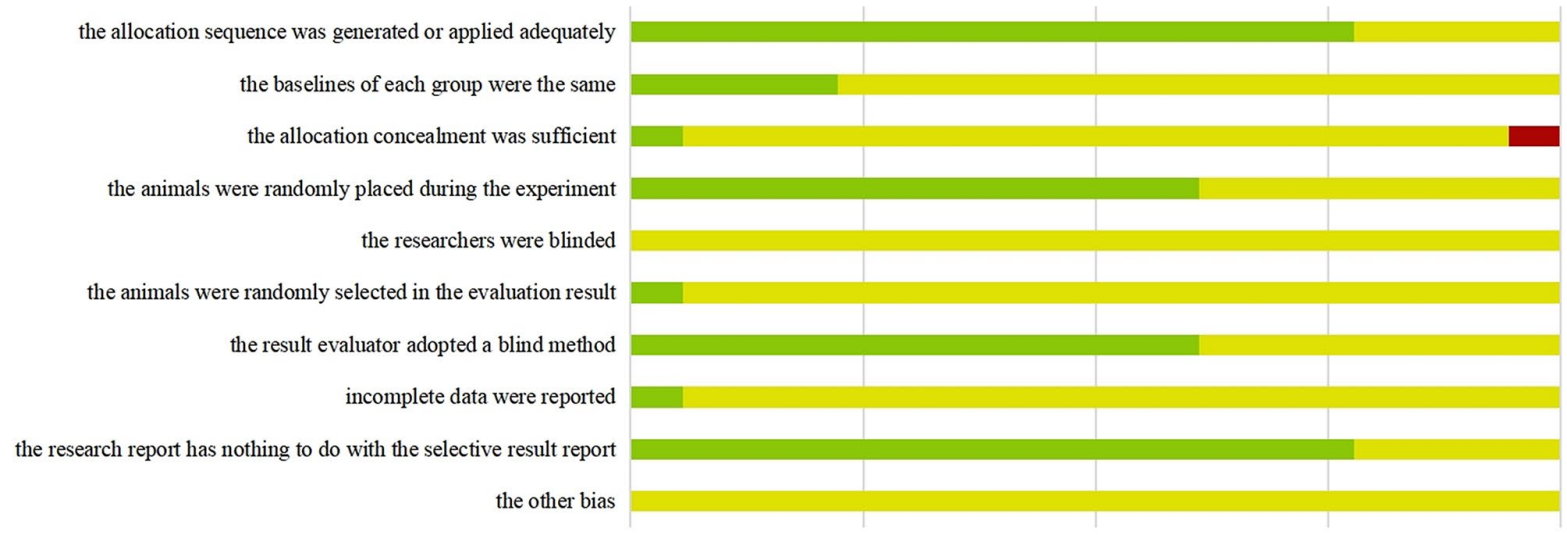
Risk of Bias Summary

Regarding the randomization items, 14 studies showed a low risk, while the remaining 4 showed an unclear risk due to the lack of reporting specific methods for randomization. Regarding baseline characteristics, 4 studies showed a low risk due to having similar baseline characteristics. One study showed a high risk in terms of concealment of allocation. All studies had an uncertain risk of blindness and other bias. Details of the methodological quality assessment are shown in Fig. 2.

### Pooled analysis of all studies

#### Pooled analysis of cardiac dysfunction and cardiac hypertrophy measures

**Fig. 3:**
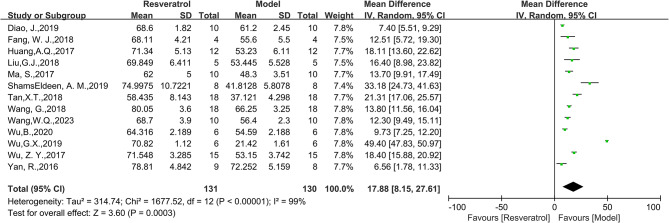
Forest plot of the effect of RES on LVEF

**Fig. 4:**
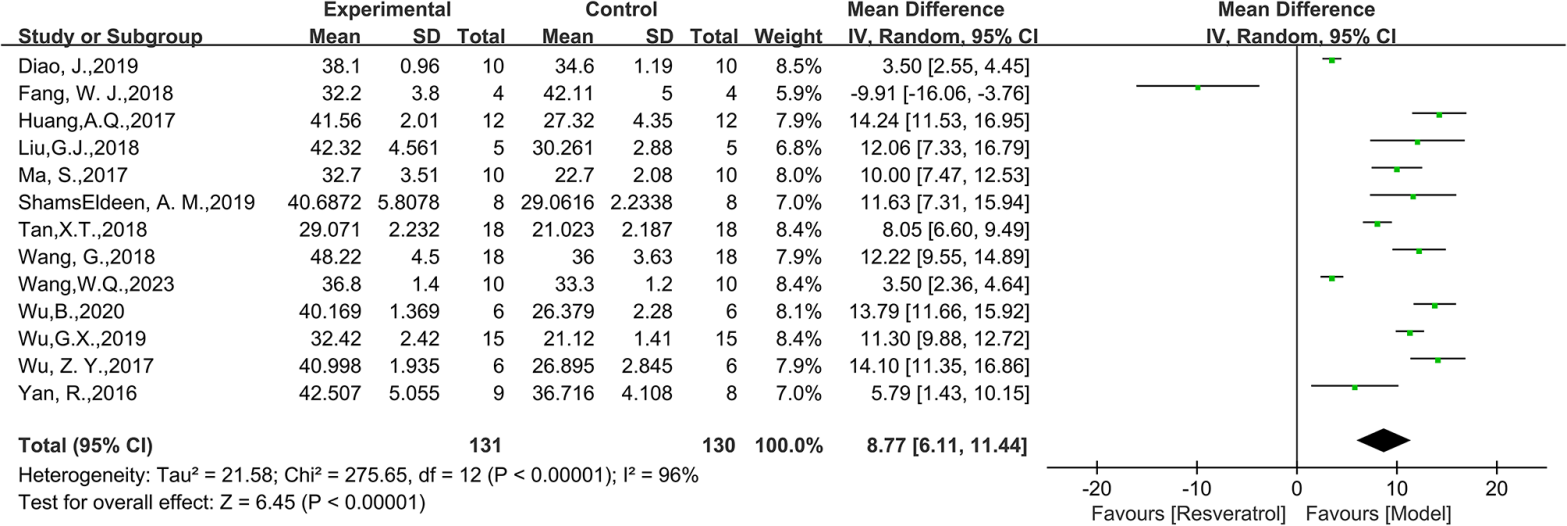
Forest plot of the effect of RES on LVFS

**Fig. 5:**
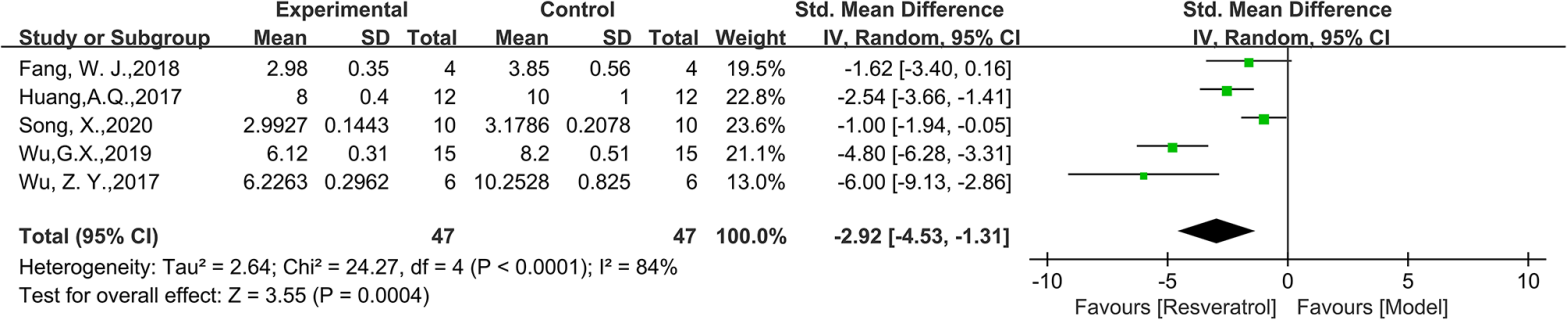
Forest plot of the effect of RES on HW/BW

Thirteen studies were included in the analysis, and the findings demonstrated a significant improvement effect of RES on LVEF index in animal models of DCM(WMD = 17.88, 95%CI: [8.15, 27.61], *P* = 0.0003, Fig. 3). There was significant heterogeneity among the studies, with an I^2^ value of 99%. Similarly, combining thirteen studies, the LVFS index in the intervention group was superior to that in the control group (WMD = 8.77, 95%CI: [6.11, 11.44], *P* < 0.0001, Fig. 4), but there was high heterogeneity between groups (I^2^ = 96%). Only one study reported E/A with an effect value of MD=-0.64, 95%CI: [-0.75,-0.53]. Five studies reported HW/BW, with 94 animals included in the analysis. The results showed that HW/BW in the RES group was significantly lower than that in the model group (SMD=-2.92, 95%CI: [-4.53,-1.31], *P*<0.0001, Fig. 5), with high heterogeneity between groups (I^2^ = 84%). We further discussed this high heterogeneity among studies in subsequent meta-regression and subgroup analysis.

#### Pooled analysis of oxidative stress measures

**Fig. 6:**
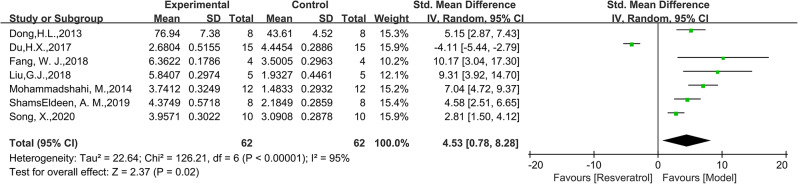
Forest plot of the effect of RES on SOD

**Fig. 7:**
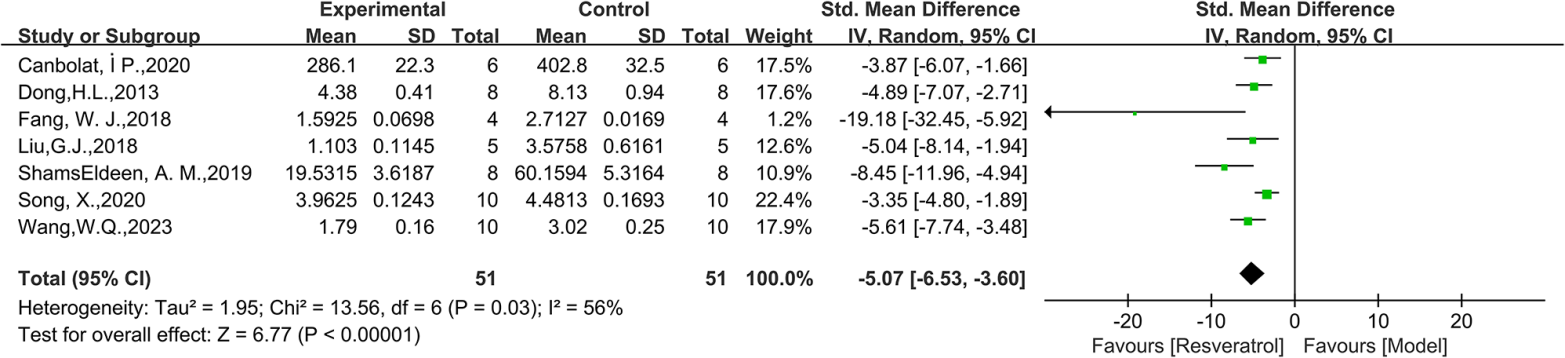
Forest plot of the effect of RES on MDA

A total of 64 animals from 7 studies were included in the analysis on SOD. The pooled analysis results showed that the SOD levels in the RES group were higher than those in the model group (SMD = 4.53, 95% CI: [0.78, 8.28], *P* = 0.002, I^2^ = 95%, Fig. 6). Research on SOD has shown high heterogeneity. When the study by Du, H.X. et al. was excluded, the heterogeneity was significantly reduced (I^2^ = 70%), suggesting that these studies may be a significant source of heterogeneity. In the subgroup analysis section, we further explored the heterogeneity. Regarding MDA, we analyzed a total of 102 samples and found that the MDA levels in the model group were higher than those in the RES group (SMD=-5.07, 95% CI: [-6.53,-3.60], *P* = 0.002, Fig. 7). There was moderate heterogeneity among the studies (I^2^ = 56%).

### Meta-regression and subgroup analysis

We conducted a regression analysis on LVEF and LVFS concerning these two primary outcome measures, but no statistically significant findings were observed.Subsequently, we further expanded our subgroup analysis based on specific factors, including sample size, animal species, animal strains, methods of establishing animal models, gender of animal models, specific administration methods, dosage, and intervention duration. However, these factors do not contribute to the heterogeneity observed in LVEF and LVFS. Results of the regression are presented in Supplementary Table 1a, b. Given that the number of studies on HW/BW and SOD is both less than 10, we did not conduct a meta-regression analysis but instead conducted subgroup analysis directly. The results indicated that for HW/BW, the route of administration may serve as a source of heterogeneity; in contrast, for SOD, factors such as animal species, strain, method of model establishment, and route of administration could contribute to heterogeneity.

### Sensitivity analysis and publication bias

Through a systematic process of elimination, we conducted a sensitivity analysis on five outcome measures, LVEF, LVFS, HW / BW, SOD, and MDA. We found that no one study had a significant impact on the results, which shows the fact that the results of our research are stable. The result is shown in Figure S1a-e.

We examined the visual funnel plots and found no significant publication bias in LVEF, HW/BW, SOD, and MDA (Figure S2a-e). The Egger’s test indicates no publication bias in LVEF (*p* = 0.023) but in LVFS (*p* = 0.13). For LVFS, we performed the shear method and added two more virtual studies, and the analysis results were consistent with the original. The results are presented in the Supplementary file 5. The GRADE grading assessment revealed that the evidence supporting LVEF, HW/BW, and MDA exhibited a moderate level of certainty, whereas the remaining factors demonstrated a low level of certainty. Table 2 provides detailed findings.

**Table 2:** Results of the grading assessment for primary outcomes

Outcomes	№ of studies	Study design	Risk of bias	Inconsistency	Indirectness	Imprecision	Other considerations	Absolute Effect	Certainty
LVEF	13	randomised trials	not serious	serious	not serious	not serious	none	-MD **17.88 higher** (8.15 higher to 27.61 higher)	⨁⨁⨁◯ Moderate
LVFS	13	randomised trials	not serious	not serious	serious	not serious	publication bias strongly suspected	-MD **8.77 higher** (6.11 higher to 11.44 higher)	⨁⨁◯◯ Low
HW/BW	5	randomised trials	not serious	serious	serious	not serious	strong association	-SMD **2.92 lower** (4.53 lower to 1.31 lower)	⨁⨁⨁◯ Moderate
SOD	7	randomised trials	not serious	serious	serious	not serious	none	SMD **4.53 higher** (0.78 higher to 8.28 higher)	⨁⨁◯◯ Low
MDA	7	randomised trials	serious	not serious	serious	not serious	strong association	SMD **5.07 lower** (6.53 lower to 3.6 lower)	⨁⨁⨁◯ Moderate

## Discussion

### Summary of the current evidence

Our study included 18 studies with 363 animals and showed that RES significantly improved cardiac function markers in the animal model of diabetic cardiomyopathy: LVEF (WMD = 17.88), LVFS (WMD = 8.77), and HW/BW(SMD=-2.92); anti-inflammatory and antioxidant markers also showed significant improvement: SOD (SMD = 4.53) and MDA(SMD=-5.07). All these results were statistically significant (*p* < 0.05). The GRADE grading assessment indicated that the certainty of LVEF, HW/BW, and MDA was moderate, while the certainty of other factors was lower. In summary, our study results suggest that RES can improve cardiac function in patients with diabetic cardiomyopathy, which may be related to mitochondrial function and its anti-inflammatory and antioxidant stress effects.

### Strengths

This study represents the first preclinical systematic review and meta-analysis, assessing the cardioprotective effects and underlying mechanisms of RES in an animal model of DCM. Firstly, we focused exclusively on a single component of RES, excluding its various derivatives, thereby providing a foundational basis for precise research and development of RES in treating diabetic cardiomyopathy. Secondly, we adhered to PRISMA guidelines throughout the meta-analysis process, ensuring that two independent investigators executed all steps to minimize errors and enhance study robustness. Thirdly, meta-regression and subgroup analyses were performed to identify sources of heterogeneity, while various analytical methods, including sensitivity analysis, were utilized to assess the stability of our findings. Lastly, we applied the GRADE tool to evaluate the quality of evidence from included animal studies; high-quality experimental data are more likely to provide reliable support for subsequent clinical investigations.

### Limitations

It is imperative to acknowledge the limitations inherent in our research. (i)The diagnosis and definition of DCM have always been controversial. In this study, when including the DCM animal model, we considered using the latest definition proposed by guidelines, which refers to impaired cardiac systolic and/or diastolic function occurring in patients with diabetes [2]. (ii) Our meta-analysis only focused on a limited number of outcome measures, including cardiac function indicators (LVEF, LVFS, HW/BW) and oxidative stress indicators (SOD, MDA). This is due to the differences in research designs that address different questions, with most studies commonly focusing on cardiac function as reflected by echocardiography. (iii)There was significant heterogeneity among studies. Meta-regression and subgroup analyses could not identify the sources of heterogeneity for LVEF and LVFS; however, the heterogeneity in HW/BW may relate to the administration route of RES, while that in SOD may stem from species, strain, model establishment methods, and administration routes used in animals. Thus, more high-quality large-sample studies are needed to validate our findings. Regarding publication bias, we conducted a trim-and-fill analysis to combine results from two dummy studies and found no statistically significant change in overall study outcomes. (iv) There may be an imbalance in the reporting of sex, as some studies did not report the sex of the included animals (50 cases, 13.7%). And it is known that the incidence of DCM is different between men and women. For diabetic patients, the probability of DCM in women is greater than that in men [41–43]. Therefore, the shortcomings of our study must be acknowledged, and this meta-analysis cannot reflect the differences in DCM between animal models of different sexes, nor whether there is a sex-specific effect of RES on DCM. (v) Due to the lack of clinical studies, we only included animal studies. However, there are differences in animal model, dose, treatment period and evaluation indicators between different studies, which may affect the direct comparison and comprehensive analysis of results. There are differences between animals and humans in physiological, biochemical and genetic aspects, so the results of animal models may not be directly applied to humans. The environmental conditions, feeding methods and experimental designs in animal experiments may not be consistent with the actual human conditions, thus affecting the applicability and repeatability of the results. In addition, the long-term effects and safety of RES have not been fully evaluated. Although short-term studies have shown benefits of RES, whether long-term use leads to adverse effects or resistance is an open question, and extrapolation from animal models to human contexts may also be challenging. (vi) Despite our best efforts to search all relevant literature, it is also worth noting that there are no negative or conflicting data. This may be due to factors such as study design, sample size, or publication bias. Therefore, we cannot completely rule out the possibility of negative results that have not yet been published or confirmed.

### Implications

To achieve a more comprehensive understanding of the role of RES in diabetic cardiomyopathy, future research must address several key areas: (i) Our study considered a limited number of outcome indicators; therefore, due to the significant heterogeneity among sources, subsequent experiments should incorporate larger sample sizes and additional indicators to validate the cardioprotective effects of RES. (ii) Given the established sex differences in disease onset, it is crucial to report sex-specific data to clarify findings. Future investigations may also benefit from exploring sex-specific drug effects on diabetic cardiomyopathy. Additionally, it may be a meaningful direction to investigate the synergistic effect of RES in combination with other therapeutic modalities, such as mesenchymal stem cell therapy [28]. (iii) While animal studies have provided preliminary evidence for the cardioprotective effects of RES in diabetic cardiomyopathy, several challenges must be addressed before these findings can be applied clinically. First, variations in animal models and experimental designs impact the reproducibility of results, necessitating the establishment of a standardized animal model based on relevant guidelines [44–46]. Second, long-term follow-up studies are crucial for thoroughly assessing the safety and efficacy of RES, focusing not only on cardiac function outcomes but also on potential side effects and overall long-term safety. Additionally, positive results from animal models must be validated in human trials, considering species differences in bioavailability when generalizing findings [47]. Finally, the duration of RES intervention is a key factor influencing clinical outcomes. Since changes in cardiac function typically occur gradually in diabetic patients, an insufficient follow-up period may hinder the detection of significant improvements [48, 49].

### Possible mechanisms

**Fig. 8:**
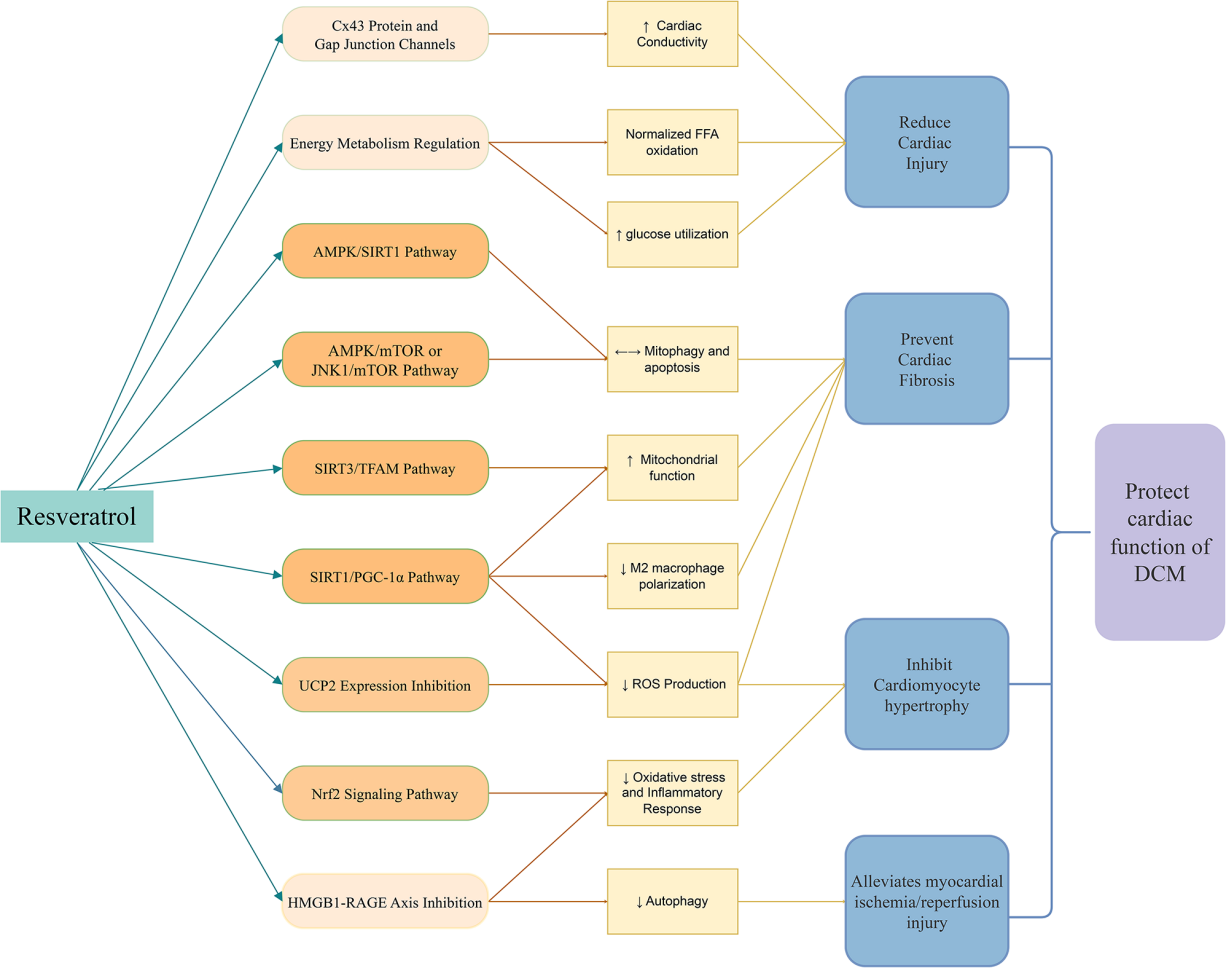
Mechanistic diagram of RES’s cardioprotective effects in an animal DCM model. Figure Legend: ↑: Improve; ↓: Reduce; ↔：Regulate

RES exhibits a range of therapeutic effects, including antioxidative, anti-inflammatory, anti-metabolic disorder, anti-apoptotic, and anti-fibrotic signaling, which contribute to the prevention and treatment of DCM [50, 51]. Furthermore, the cardioprotective effects of RES on DCM are suggested to involve multiple pathways, including the SIRT1/PGC-1α, SIRT3/TFAM, AMPK/mTOR, JNK1/mTOR, Nrf2, Cx43 protein and gap junction channels, UCP2 expression, HMGB1-RAGE axis, and energy metabolism regulation [52–67]. We have encapsulated this understanding in a mechanism diagram (Fig. 8).

Based on these pathways, studies have shown that RES inhibits cardiomyocyte hypertrophy and improves cardiomyocyte fibrosis by up-regulating SIRT1 expression, reducing reactive oxygen species (ROS) accumulation, and regulating mitochondrial homeostasis through SIRT1/PGC-1α pathway [52, 53]. Moreover, RES activates SIRT and inhibits the polarization of M2 macrophages stimulated by advanced glycation end products (AGEs) [54]. This is complemented by the findings of Pankaj K. Bagul et al., who noted that SIRT-3 deficiency decreases the activity of mitochondrial transcription factor A (TFAM), while RES regulates TFAM’s acetylation state and improves cardiac mitochondrial function [55].

Shifting focus to AMPK (AMP-activated protein kinase), it plays a role in various aspects of mitochondrial homeostasis, such as mitophagy, thereby affecting mitochondrial health [56]. Studies have shown that RES can delay fibrosis in DCMby regulating the mitophagy response through the AMPK/SIRT1-mediated IRE1α/PTEN-induced PINK signaling pathway [57, 58]. Additionally, Kui Xu et al. observed that RES can activate AMPK and JNK1, inhibit mTOR and its downstream effects, regulate mitophagy and apoptosis, and thus exert a protective effect on DCM cardiomyocytes [59].

Nrf2, which is linked to increased inflammatory response and oxidative stress in diabetic cardiomyopathy [60], is also targeted by RES. Experimental evidence confirms that RES enhances Nrf2 expression and transcription, preventing DCM through anti-inflammatory and antioxidant effects [61].

Furthermore, research indicates that RES diminishes the upregulation of Cx43 and apoptosis in H9c2 cells caused by high glucose, potentially enhancing the electrical conductivity of the diabetic heart and stimulating the autophagy signaling pathway [62, 63].

Expanding on the protective mechanisms, Han Wu’s experiments on STZ-induced diabetic mice revealed that RES prevents myocardial fibrosis by regulating the bone morphogenetic protein signaling pathway, inhibiting UCP2 expression, and reducing ROS production [64].

Additionally, studies have shown that RES may alleviate HMGB1-mediated myocardial ischemia-reperfusion injury in diabetic rats by inhibiting HMGB1 or RAGE-mediated autophagy, reducing oxidative stress and inflammation [65, 66].

Beyond these molecular signaling pathways, energy regulation and metabolism are also key mechanisms by which RES combats DCM. Studies indicate that RES can normalize free fatty acid (FFA) oxidation in diabetic conditions, enhance glucose utilization, and regulate oxidative stress biomarkers, thereby reducing myocardial damage [67, 68].

## Conclusion

Our research findings suggest that RES may possess the potential to protect cardiac function in animal models with diabetic cardiomyopathy, possibly due to its anti-inflammatory and antioxidant properties. However, further clinical trials are needed to explore its clinical efficacy.

## Electronic supplementary material

Below is the link to the electronic supplementary material.

Supplementary Material 1: The PRISMA checklist

Supplementary Material 2: Search formula

Supplementary Material 3: Results of the regression and subgroup analyses of LVEF, Results of the regression and subgroup analyses of LVFS

Supplementary Material 4: The Sensitivity analysis for LVEF, The Sensitivity analysis for LVFS, The Sensitivity analysis for HW/BW, The Sensitivity analysis for SOD, The Sensitivity analysis for MDA, The funnel plot of LVEF, The funnel plot of LVFS, The funnel plot of HW/BW, The funnel plot of SOD, The funnel plot of MDA

Supplementary Material 5: The results of shear compensation for LVFS

## Data Availability

The article and its supplementary files contain the datasets that substantiate the findings mentioned in this paper.

## Abbreviations

BW: Body weight
DCM: Diabetic ardiomyopathy
DM: Diabetes Mellitus
ESC: European Society of Cardiology
FVB: Friend Virus B NIH Jackson
HW: Heart weight
LVEF: Left Ventricular Ejection Fraction
LVFS: Left Ventricular Fractional Shortening Rate
MAD: Malonaldehyde
RES: Resveratrol
ROS: Reactive oxygen species
SD: Sprague Dawley
SMD: Standardized mean difference
SOD: Superoxide Dismutase
STZ: Streptozocin
SYRCLE: Systematic Review Center for Laboratory animal Experimentation
WMD: Weighted mean difference
