# Supplementary material for: Preclinical evidence and possible mechanisms of cardioprotective effects of resveratrol in diabetic cardiomyopathy: a systematic review and meta-analysis
Source: Diabetol Metab Syndr. 2024 Nov 17;16:275. doi: 10.1186/s13098-024-01512-8 (PMC11572515; doi:10.1186/s13098-024-01512-8)
Supplement: Supplementary file 2 — Supplementary Material 2: Search formula [file 13098_2024_1512_MOESM2_ESM.docx]

**Search formula**

1. PubMed:

("resveratrol"[Title/Abstract] OR "Vitis vinifera"[Title/Abstract]) AND (("Diabetic cardiomyopathy"[Title/Abstract] OR "Diabetic Cardiomyopathies"[Title/Abstract]))

2. Embase:

('resveratrol':ti,ab,kw OR 'vitis vinifera':ti,ab,kw) AND ('diabetic cardiomyopathy':ti,ab,kw OR 'diabetic cardiomyopathies':ti,ab,kw)

3. Web of Science:

"resveratrol" OR "Vitis vinifera" (Topic) AND "Diabetic cardiomyopathy" OR "Diabetic Cardiomyopathies" (Topic)

4. the Cochrane Library:

"resveratrol" OR "Vitis vinifera" in Title Abstract Keyword AND "Diabetic cardiomyopathy" OR "Diabetic Cardiomyopathies" in Title Abstract Keyword - (Word variations have been searched)

5. Chinese National Knowledge Infrastructure:

Topic: bai li lu chun + 'bai li lu chun (res)' + bai li lu chun gan + bai li lu chun ganyu + bai li lu chun yanshengwu + bu chong bai li lu chun) AND (Topic: tang niao bing xin ji bing + tang niao bing xin ji bing da shu + 'tang niao bing xin ji bing (dcm)' + tang niao bing xin ji bing bian + tang niao bing xin ji bing mo xing + 2 xing tang niao bing xin ji bing)

In the retrieval of cnki, the search terms recommended by database intelligence are used."bai li lu chun" (meaning RES) and "tang niao bing xin ji bing" (meaning Diabetic cardiomyopathy);“da shu” (meaning rats);“mo xing” (meaning model);“2 xing” (meaning type 2); “tang niao bing xin ji bing bian”(meaning Diabetic cardiomyopathy); “gan” (meaning glycoside); “gan yu”(meaning intervention); “yan sheng wu”(meaning derivatives);“bu chong” (meaning supplementation).

6. Chinese Biomedical Literature Database

"bai li lu chun "[common field: smart] AND" tang niao bing xin ji bing "[common field: smart]

"bai li lu chun" (meaning RES) and "tang niao bing xin ji bing" (meaning Diabetic cardiomyopathy)

7. Chinese VIP Database:

Title or key words = bai li lu chun * tang niao bing xin ji bing

"bai li lu chun" (meaning RES) and "tang niao bing xin ji bing" (meaning Diabetic cardiomyopathy)

8. WanFang Database:

(Subject: bai li lu chun) and Subject: tang niao bing xin ji bing)

"bai li lu chun" (meaning RES) and "tang niao bing xin ji bing" (meaning Diabetic cardiomyopathy)
