# Supplementary material for: Preclinical evidence and possible mechanisms of cardioprotective effects of resveratrol in diabetic cardiomyopathy: a systematic review and meta-analysis
Source: Diabetol Metab Syndr. 2024 Nov 17;16:275. doi: 10.1186/s13098-024-01512-8 (PMC11572515; doi:10.1186/s13098-024-01512-8)
Supplement: Supplementary file 3 — Supplementary Material 3: Results of the regression and subgroup analyses of LVEF, Results of the regression and subgroup analyses of LVFS [file 13098_2024_1512_MOESM3_ESM.docx]

**Supplementary Table 1a.** Results of the regression and subgroup analyses of LVEF

| _es | Coefficient | Std. err. | t | P>\|t\| | [95% conf. | interval] |
| --- | --- | --- | --- | --- | --- | --- |
| sample | 1.404899 | 1.039047 | 1.35 | 0.248 | -1.479957 | 4.289755 |
| species | 2.454821 | 20.74397 | 0.12 | 0.912 | -55.13966 | 60.0493 |
| animal strains | 3.372317 | 5.782184 | 0.58 | 0.591 | -12.6816 | 19.42623 |
| modeling approach | -6.477791 | 20.44092 | -0.32 | 0.767 | -63.23087 | 50.27529 |
| gender | -19.89145 | 26.92737 | -0.74 | 0.501 | -94.65381 | 54.87092 |
| intervention approach | 4.54321 | 6.444741 | 0.7 | 0.52 | -13.35026 | 22.43668 |
| intervention dose | 23.28154 | 20.87767 | 1.12 | 0.327 | -34.68415 | 81.24724 |
| intervention duration | -6.663957 | 11.13287 | -0.6 | 0.582 | -37.57377 | 24.24586 |
| _cons | -9.777661 | 72.18431 | -0.14 | 0.899 | -210.1934 | 190.6381 |

**Supplementary Table 1b.** Results of the regression and subgroup analyses of LVFS

| _es | Coefficient | Std. err. | t | P>\|t\| | [95% conf. | interval] |
| --- | --- | --- | --- | --- | --- | --- |
| sample | 1.404899 | 1.039047 | 1.35 | 0.248 | -1.479957 | 4.289755 |
| species | 2.454821 | 20.74397 | 0.12 | 0.912 | -55.13966 | 60.0493 |
| animal strains | 3.372317 | 5.782184 | 0.58 | 0.591 | -12.6816 | 19.42623 |
| modeling approach | -6.477791 | 20.44092 | -0.32 | 0.767 | -63.23087 | 50.27529 |
| gender | -19.89145 | 26.92737 | -0.74 | 0.501 | -94.65381 | 54.87092 |
| intervention approach | 4.54321 | 6.444741 | 0.7 | 0.52 | -13.35026 | 22.43668 |
| intervention dose | 23.28154 | 20.87767 | 1.12 | 0.327 | -34.68415 | 81.24724 |
| intervention duration | -6.663957 | 11.13287 | -0.6 | 0.582 | -37.57377 | 24.24586 |
| _cons | -9.777661 | 72.18431 | -0.14 | 0.899 | -210.1934 | 190.6381 |
