# Supplementary material for: Preclinical evidence and possible mechanisms of cardioprotective effects of resveratrol in diabetic cardiomyopathy: a systematic review and meta-analysis
Source: Diabetol Metab Syndr. 2024 Nov 17;16:275. doi: 10.1186/s13098-024-01512-8 (PMC11572515; doi:10.1186/s13098-024-01512-8)
Supplement: Supplementary file 4 — Supplementary Material 4: The Sensitivity analysis for LVEF, The Sensitivity analysis for LVFS, The Sensitivity analysis for HW/BW, The Sensitivity analysis for SOD, The Sensitivity analysis for MDA, The funnel plot of LVEF, The funnel plot of LVFS, The funnel plot of HW/BW, The funnel plot of SOD, The funnel plot of MDA [file 13098_2024_1512_MOESM4_ESM.docx]

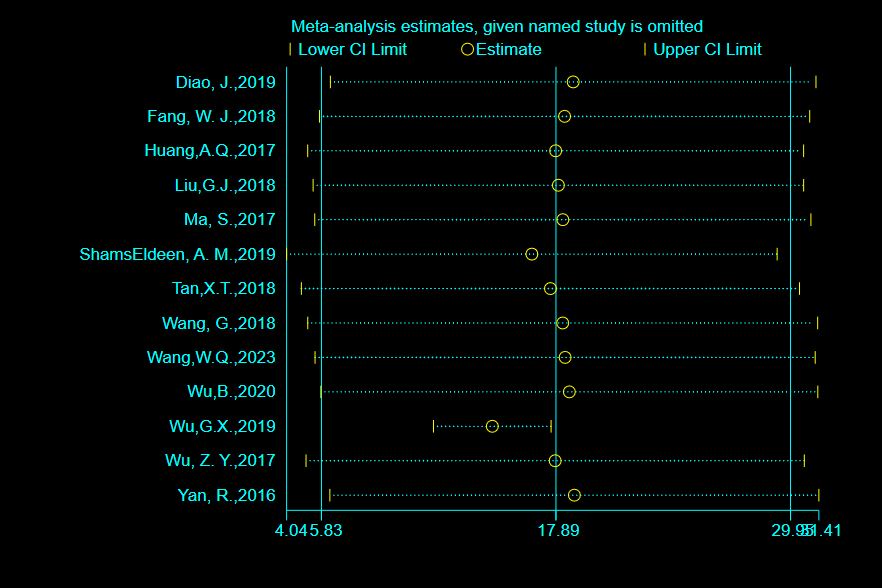


Fig. S1a.The Sensitivity analysis for LVEF


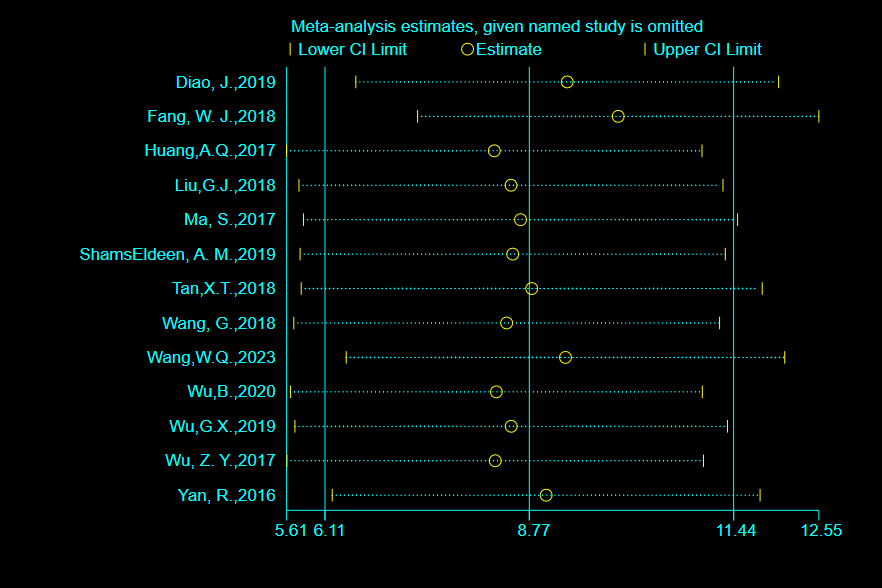


Fig. S1b.The Sensitivity analysis for LVFS


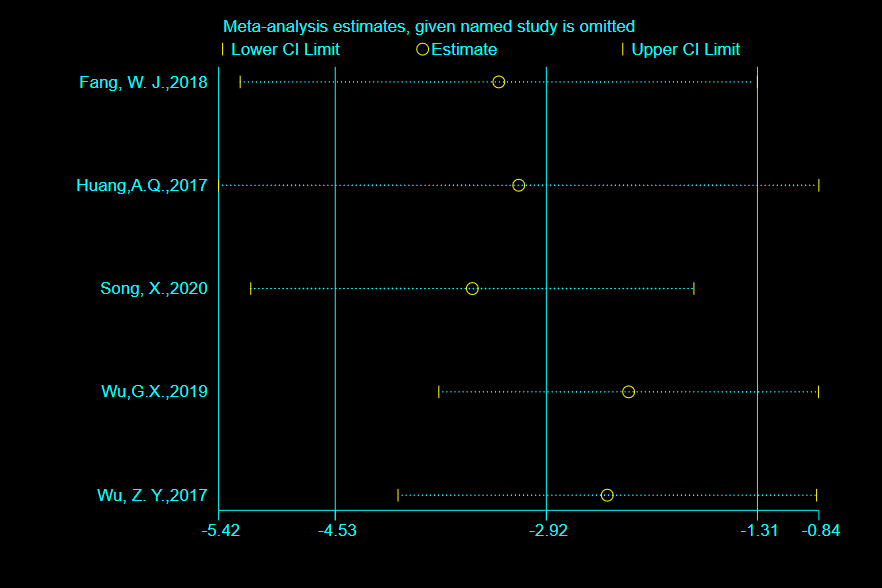


Fig. S1c.The Sensitivity analysis for HW/BW


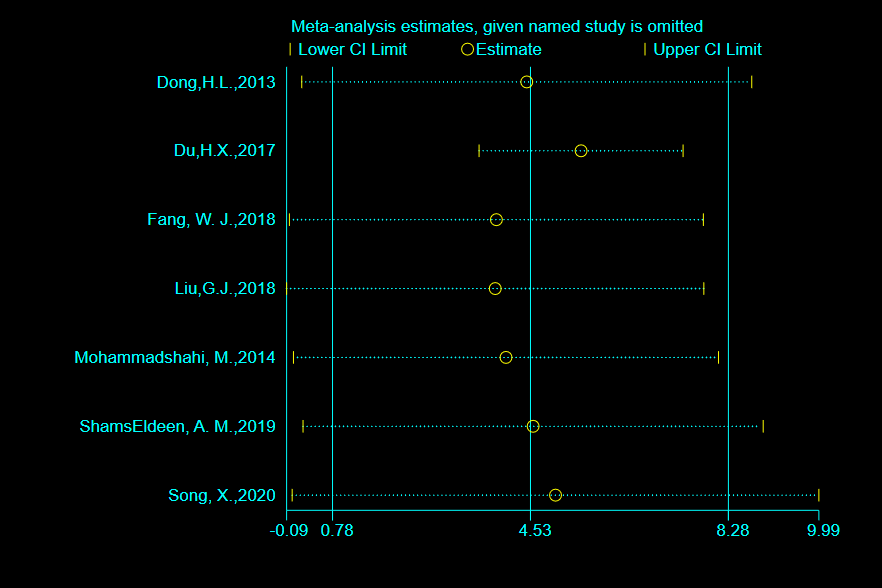


Fig.S1d.The Sensitivity analysis for SOD


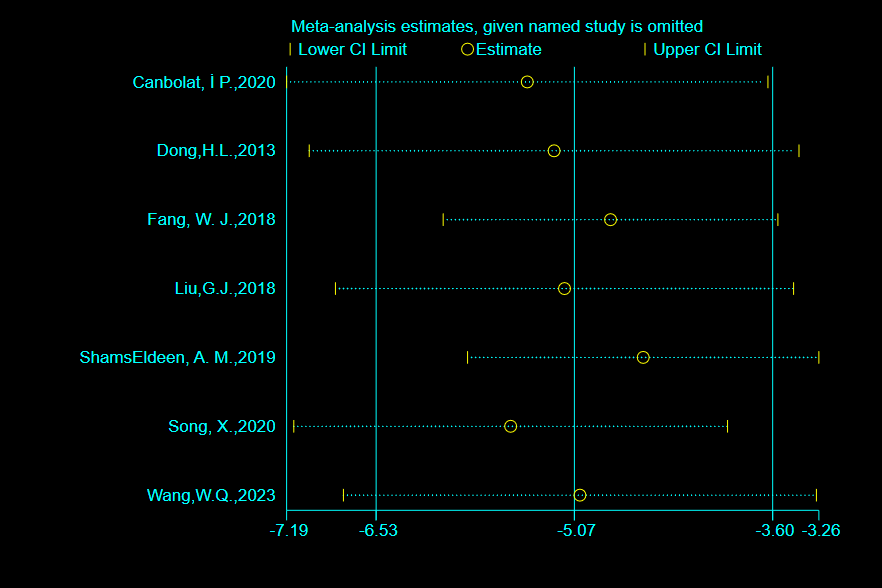


Fig.S1e.The Sensitivity analysis for MDA


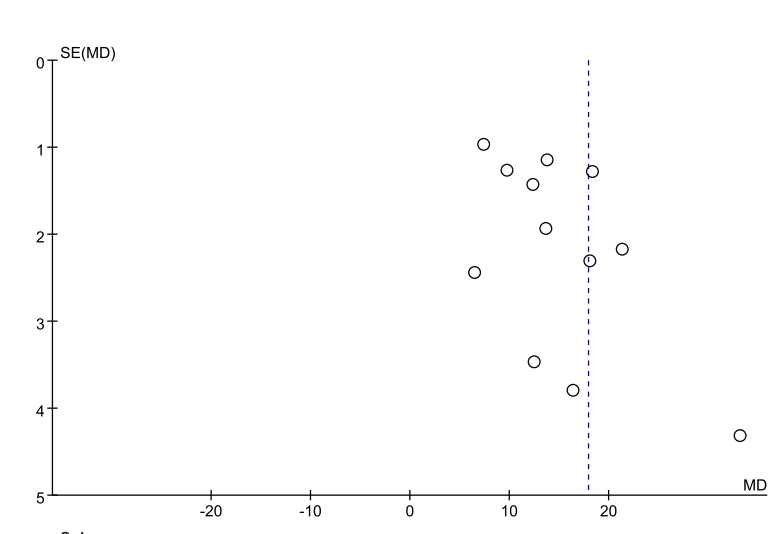


Fig. S2a.The funnel plot of LVEF


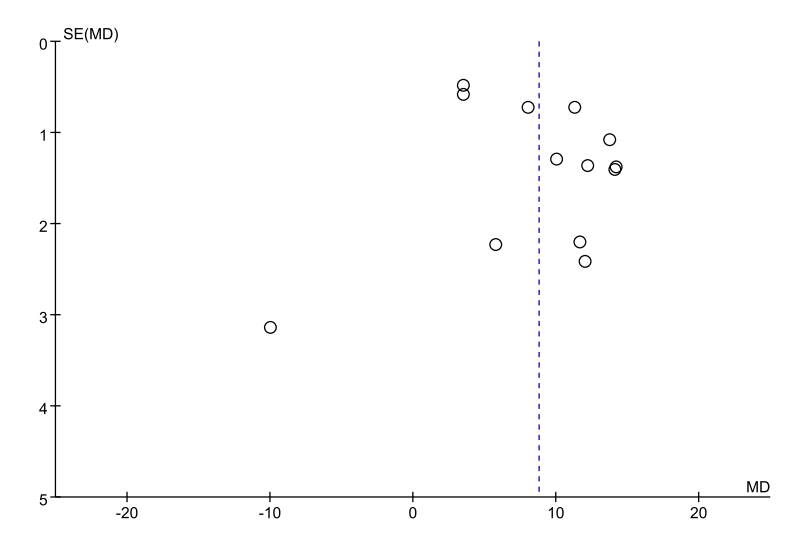


Fig. S2b.The funnel plot of LVFS


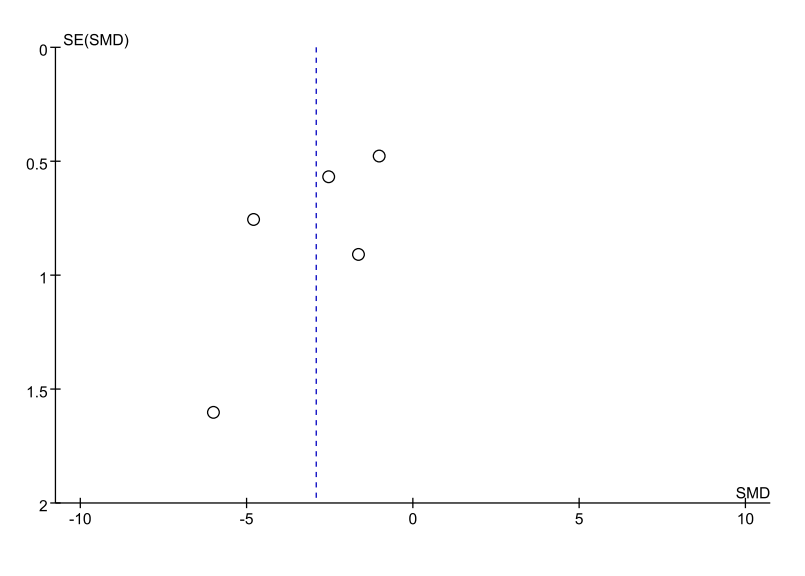


Fig. S2c.The funnel plot of HW/BW


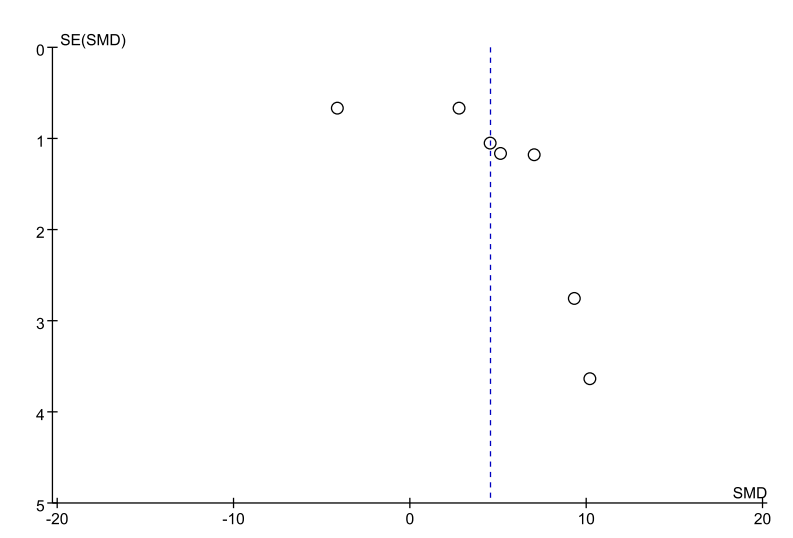


Fig. S2d.The funnel plot of SOD


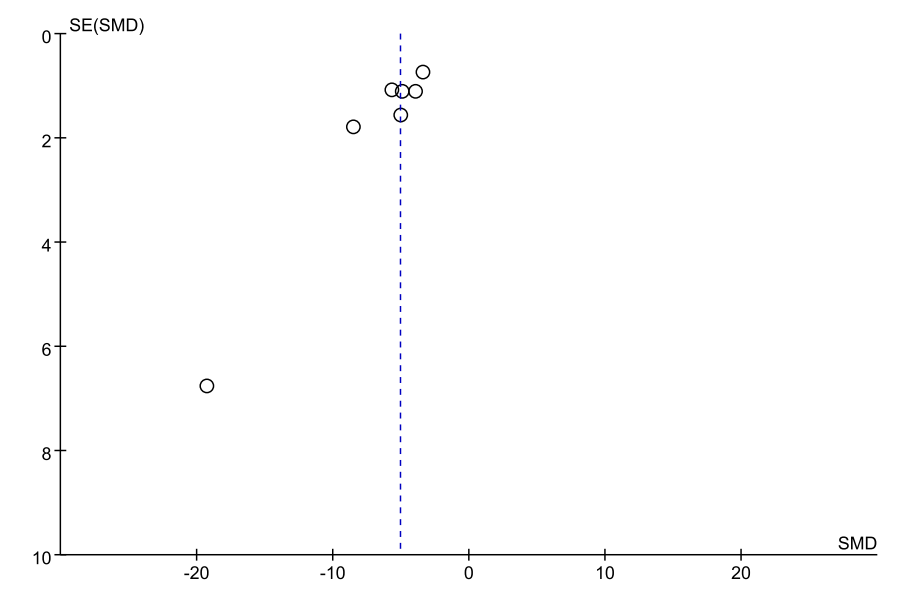


Fig. S2e.The funnel plot of MDA
