# Supplementary material for: Preclinical evidence and possible mechanisms of cardioprotective effects of resveratrol in diabetic cardiomyopathy: a systematic review and meta-analysis
Source: Diabetol Metab Syndr. 2024 Nov 17;16:275. doi: 10.1186/s13098-024-01512-8 (PMC11572515; doi:10.1186/s13098-024-01512-8)
Supplement: Supplementary file 5 — Supplementary Material 5: The results of shear compensation for LVFS [file 13098_2024_1512_MOESM5_ESM.docx]

**· The results of shear compensation for LVFS**

To address the issue of publication bias in LVFS, we conducted a trim-and-fill analysis to assess its impact on result reliability. Following linear iteration, we incorporated two virtual studies alongside the original 13 studies. The final meta-analysis results, as depicted in Figure X, consistently demonstrated that the intervention group exhibited superior effects on LVFS compared to the control group.
